# Quantitative Ultrasound: Measurement Considerations for the Assessment of Muscular Dystrophy and Sarcopenia

**DOI:** 10.3389/fnagi.2014.00172

**Published:** 2014-07-14

**Authors:** Michael O. Harris-Love, Reza Monfaredi, Catheeja Ismail, Marc R. Blackman, Kevin Cleary

**Affiliations:** ^1^Geriatrics and Extended Care Service, Veterans Affairs Medical Center, Washington, DC, USA; ^2^Research Service, Veterans Affairs Medical Center, Washington, DC, USA; ^3^Department of Exercise Science, School of Public Health and Health Services, The George Washington University, Washington, DC, USA; ^4^Sheikh Zayed Institute for Pediatric Surgical Innovation, Children’s National Hospital, Washington, DC, USA; ^5^Department of Medicine, School of Medicine and Health Sciences, The George Washington University, Washington, DC, USA

**Keywords:** ultrasound, muscular dystrophy, sarcopenia, screening, assessment, skeletal muscle

## Introduction

Diagnostic musculoskeletal ultrasound is a non-invasive, low-cost, imaging modality that may be used to characterize normal and pathological muscle tissue. Sonography has been long proposed as a method of assessing muscle damage due to neuromuscular diseases such as muscular dystrophy (Reimers et al., 1996), and more recently, changes in body and tissue composition associated with muscle wasting disorders such as sarcopenia (Pillen and van Alfen, 2011). The use of quantitative ultrasound as an adjunct diagnostic procedure has different technical challenges than the traditional use of ultrasound in clinical medicine. Examiner-dependent technique and variation are critical considerations when assessing the presence of muscle atrophy via tissue dimension estimates using muscle thickness measures, or when quantifying pathological changes in muscle quality via estimates of echointensity using grayscale analysis. Understanding both the promise of quantitative ultrasound as an assessment tool for muscle disorders and the known threats to measurement validity may foster greater adoption of this imaging modality in the management of muscular dystrophy and sarcopenia.

## Diagnostic Ultrasound Utilization in the Management of Muscular Dystrophy and Sarcopenia: Similarities and Differences in Approach

### Common morphological features

Muscular dystrophy is a broad term that encompasses a disease group marked by progressive skeletal muscle weakness, atrophy, and myofiber degeneration with heterogeneous genetic etiologies that include epigenetic, monogenic, and repeat expansion abnormalities (Leung and Wagner, 2013). Muscular dystrophy affects both children and adults, which reflects its wide ranging phenotypic expression. In contrast, many investigators regard sarcopenia as an age-related condition denoted by a loss of lean body mass (LBM) with diminished muscle strength or functional performance (Newman et al., 2003; Cruz-Jentoft et al., 2010; Morley et al., 2011). However, it is important to note that a more expansive view of an “all cause” designation for muscle impairment, i.e., myopenia or skeletal muscle function deficit, has been recognized as an approach to nosology that may serve to limit the confounding effect of incongruent definitions, and facilitate the discovery of linkages among apparently disparate forms of muscle dysfunction (Fearon et al., 2011; von Haehling et al., 2012; Correa-de-Araujo and Hadley, 2014). Muscular dystrophy is recognized as a group of diseases, whereas sarcopenia is widely regarded as a geriatric syndrome. Nevertheless, it has been proposed that these two muscle disorders have some common morphological features such as the centralization of sarcolemic nuclei, atrophic groups of muscle fibers, and excessive variation of muscle fiber size (Edström et al., 2007; Malatesta, 2012). Furthermore, individuals with muscular dystrophy or sarcopenia may exhibit excessive intramuscular adipose tissue, intramyocellular triglyceride levels, and non-contractile infiltrates (Pillen et al., 2003; Miljkovic-Gacic et al., 2008; Jansen et al., 2012). Therefore, sonographic measures of echointensity for the purpose of tissue composition estimates, and digital caliper measures of tissue dimensions to assess muscle atrophy are both key elements of the ultrasound assessment of muscular dystrophy and sarcopenia (Pillen and van Alfen, 2011; Tieleman et al., 2012; Janssen et al., 2014).

### Condition-specific approach to diagnostic ultrasound

In muscular dystrophies, quantitative ultrasound has been frequently proposed for Duchenne muscular dystrophy (DMD) (Pillen et al., 2003; Scholten et al., 2003; Jansen et al., 2012). The measurement of echointensity using grayscale histogram analysis has been used as a proxy measure for the increased non-contractile features associated with the pathologic muscle changes that may result in DMD. Jansen et al. (2012) reported that echointensity values were significantly associated with ambulation status, functional performance, and hand-held dynamometry peak force values in children with DMD. The observed standardized response mean (SRM) for their echointensity values over a 1-year period was 0.77 for their summed scores, with the lower extremities (SRM = 0.79–0.89) exhibiting greater responsiveness in comparison with the upper extremities (SRM = 0.35–0.36). Additionally, Pillen et al. (2007) have shown that echointensity and muscle thickness values have diagnostic utility as supported by the demonstrated discriminative validity of quantitative ultrasound among children suspected of having a neuromuscular disorder. Moreover, in some instances, M-mode ultrasound may have advantages over electromyography for the assessment of muscle fasciculations, which is a clinical feature of some forms of muscular dystrophy and myopathy (Walker et al., 1990; Scheel et al., 1997; Pillen and van Alfen, 2011).

The use of quantitative ultrasound for the assessment of sarcopenia has been previously proposed (Pillen and van Alfen, 2011), but this approach has not been embraced by the largest international societies that issue position stands and consensus statements regarding the diagnostic criteria for sarcopenia (Cruz-Jentoft et al., 2010; Morley et al., 2011; Studenski et al., 2014). Less developmental work has been completed concerning the use of ultrasound in the assessment of age-related muscle changes in comparison to more well-known approaches involving dual-energy X-ray absorptiometry (DXA), computed tomography (CT), or magnetic resonance imaging (MRI), bioelectrical impedance analysis (BIA), and other anthropometric-based methods. However, important foundational research concerning the use of ultrasound to determine body composition has been completed, which merits the attention of clinicians and investigators interested in the diagnosis and management of sarcopenia. Previous study findings suggest that ultrasound LBM estimates have concurrent validity with MRI (Abe et al., 1994) and hydrodensitometry (Sanada et al., 2006) in Japanese adults. In the study by Abe et al. (1994), a nine-site anatomical model for ultrasound-derived LBM displayed moderate to strong relationships with MRI muscle density values (*r* = 0.83–0.96 in men, *r* = 0.53–0.91 in women, *n* = 72, 18–61 years of age, *p* < 0.05). Similar approaches to quantitative ultrasound have also been successfully employed to estimate body fat in adults (Pineau et al., 2007, 2009; Wagner, 2013). An emergent view concerning the effect of the age-related increase in intramuscular adipose tissue on muscle performance and lower extremity impairments (Goodpaster et al., 2001) has important implications concerning the optimal approach to the sarcopenia diagnosis. The ultrasound measurement of echointensity and muscle thickness may provide a more comprehensive method of assessing LBM that accounts for both muscle quantity and muscle quality.

## Examiner-Dependent Factors That Affect the Ultrasound Image: Force and Angle

### Examiner-dependent factors and quantitative ultrasound

Investigators have demonstrated that ultrasound is a reliable tool between raters and examination sessions (Hides et al., 2007), and with a variety of muscle groups (Bemben, 2002; O’Sullivan et al., 2007; Cheng et al., 2012; Temes et al., 2014). Nonetheless, it is important to recognize that ultrasound has a degree of examiner-dependency that is higher in comparison with other modes of imaging such as DXA, CT scanning, or MRI. Consequently, extending the findings of research reports on measurement reliability to typical clinical environments should be done with a degree of caution. The orientation of the sound transducer relative to the body surface and the compressive or shear stress on tissue through the force exerted by the examiner can alter tissue dimensions and echointensity. Ishida and Watanabe (2012) have cited the influence of compressive stress exerted by the examiner with the ultrasound transducer as a potential source of error in the assessment of abdominal muscle thickness. Also, it has been noted that alterations in the sound transducer orientation may result in measurement error when estimating muscle size and ultrastructure features such as pennation angle (Herbert and Gandevia, 1995; Dupont et al., 2001). Whittaker et al. (2009) reported that no significant changes in transversus abdominis thickness measurements were observed when sound transducer rotation was <9° and cranial/caudal tilting was <5°. The aforementioned observations suggest that structured methods of training and standardized procedures may benefit the clinical application of the ultrasound imaging to obtain quantitative measures.

### Feedback-augmented quantitative ultrasound

**Figure 1:**
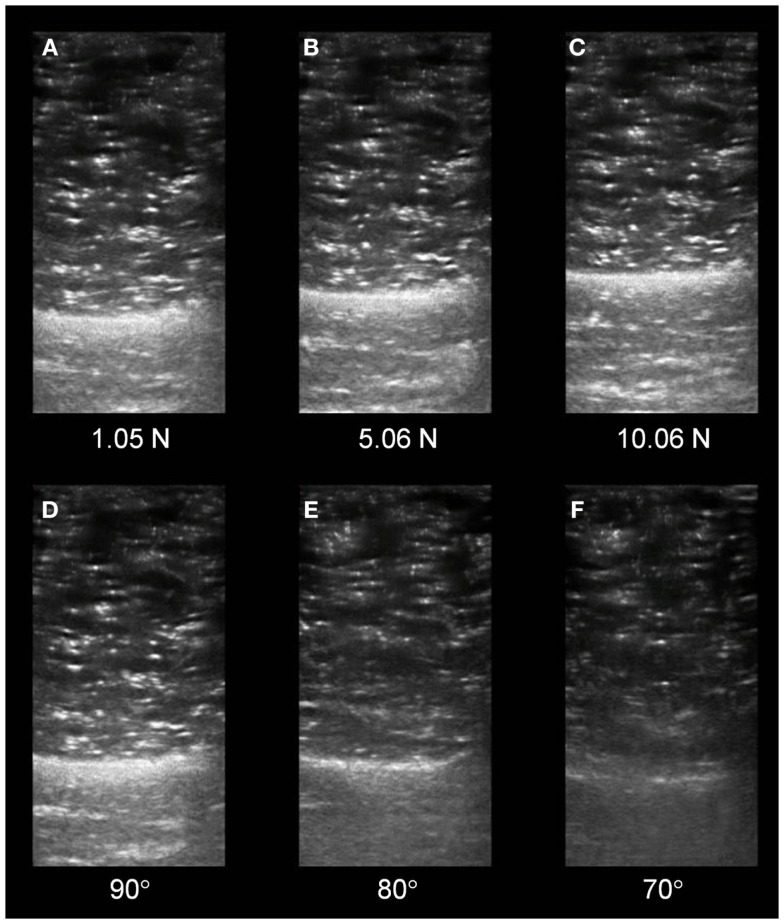
**Changes in serial sonographic image characteristics based on examiner force and sound transducer orientation**. **(A–C)** Depict transverse views of a muscle tissue mimetic phantom with a progressive magnitude of stress imposed on the phantom surface by the sound transducer. The material deformation (thickness, centimeter) secondary to the stress progression was as follows: **(A)** 3.78 cm, **(B)** 3.45 cm, and **(C)** 3.21 cm. **(D–F)** Depict similar sonographic views as the preceding panels. The echointensity observed in the serial images is based on a progressively increasing cranial/caudal tilt angle of the sound transducer applied to the phantom surface. The changes in echointensity (grayscale, unitless, 0–255) secondary to the angle progression were as follows: **(D)** 56.64, **(E)** 48.10, and **(F)** 36.90. (All images were acquired using a 6 MHz linear array sound transducer and a muscle mimetic phantom with anechoic gel via automated image capture by the Kuka LWA robot.)

Our group is exploring the use of real-time augmented feedback for quantitative ultrasound imaging. Real-time, free-hand, diagnostic ultrasound inherently features visual feedback of the region of interest (ROI) during an imaging procedure. However, this mode of feedback alone may be insufficient to control factors related to examiner force and sound transducer orientation. The serial ultrasound image exemplar depicted in the Figure 1 illustrates the effect of compressive stress and cranial/caudal tilting of the sound transducer on material characteristics within the ROI. The B-mode images were obtained with a portable ultrasound unit (SonoSite Titan M-Turbo) using a 6 MHz linear array sound transducer with a custom interface featuring a load cell (FC22 Compression Load Cell; 0–44.48 ± 0.45 N). Automated image acquisition and sound transducer positioning were performed with the Kuka light weight arm (LWA) robot (7 degrees of freedom; motion error, ±0.05 mm) to attain uniform force and angle targets. The scanned material was a custom calibration phantom designed as a skeletal muscle mimetic (i.e., anechoic gel, 15 kPa; speed of sound, 1540 m/s; attenuation, 0.1 dB/cm/MHz; CIRS, Inc.). A single examiner performed the digital caliper measures and echointensity was estimated via grayscale histogram analysis using a method adapted from Scholten et al. (2003) and Ismail et al. (2014). Our attained measurement values are consistent with the observations of Ishida and Watanabe (2012) regarding the negative effect of excessive compressive stress on material dimensions. Additionally, the serial images illustrate that progressive shifts in cranial/caudal tilting of 10° resulted in a >15% decrease in echointensity. While our use of automated image capture and a muscle mimetic phantom are primarily for testing and training purposes, the custom feedback-augmented sound transducer interface is portable and may used to guide free-hand ultrasound imaging.

## Adoption of Quantitative Ultrasound in the Assessment of Muscular Dystrophy and Sarcopenia

Qualitative diagnostic ultrasound is often focused on the identification and subjective description of an anatomical structure or pathological tissue anomaly. Sonographers frequently use variable levels of force and sound transducer angle to obtain images of deep structures with sufficient resolution for clinical use. In contrast, quantitative ultrasound is generally dependent on the examiner exerting minimal stress on the tissue or structure of interest, and using consistent transducer orientation to attain reliable serial or comparative measures. Therefore, the use of calibration phantoms and force-feedback-augmented ultrasound may be viable methods of providing operator training and aiding real-time ultrasound measurement consistency.

The constraints associated with quantitative ultrasound tend to limit this form of assessment to superficial tissues (Pillen and van Alfen, 2011), and additional normative datasets are needed to facilitate the interpretation of cross-sectional data – particularly for older adults with sarcopenia. Also, while muscle thickness measures may be fairly uniform across ultrasound platforms, echointensity values require a correction factor for comparisons involving different ultrasound machines (Zaidman et al., 2010). Notably, qualitative ultrasound has an important role in the management of neuromuscular disease as variable examiner-force and transducer orientation is needed to locate focal areas of hyperechoic tissue for potential biopsy sites (Pillen et al., 2007). Despite these limitations and contingencies, quantitative ultrasound remains a useful clinical and research imaging option to characterize skeletal muscle in muscular dystrophy and sarcopenia. This imaging modality provides a non-invasive, inexpensive method to assess muscle morphology and estimate tissue and body composition without the use of ionizing radiation. Attention to factors such as imaging site location, patient positioning, examiner training, the standardization of specific assessment techniques, and the optimal use of imaging feedback may aid the wider adoption of sonography for the management of muscle disorders.

## Conflict of Interest Statement

The authors declare that the research was conducted in the absence of any commercial or financial relationships that could be construed as a potential conflict of interest.
